# Community phylogeny and spatial scale affect phylogenetic diversity metrics in a species‐rich rainforest in Borneo

**DOI:** 10.1002/ece3.9536

**Published:** 2022-11-22

**Authors:** Seiya Okuno, Tingting Yin, Satoshi Nanami, Shuhei Matsuyama, Koichi Kamiya, Sylvester Tan, Stuart J. Davies, Mohizah Mohamad, Takuo Yamakura, Akira Itoh

**Affiliations:** ^1^ Graduate School of Science Osaka City University Osaka Japan; ^2^ Graduate School of Science Osaka Metropolitan University Osaka Japan; ^3^ College of Agriculture, Food and Environmental Sciences Rakuno Gakuen University Ebetsu Japan; ^4^ Graduate School of Agriculture Ehime University Matsuyama Japan; ^5^ Global Earth Observatory (ForestGEO) Smithsonian Tropical Research Institute Washington USA; ^6^ Forest Department Sarawak Kuching Sarawak Malaysia

**Keywords:** community phylogeny, DNA barcoding, South‐East Asia, tropical rainforest

## Abstract

Community phylogenetic analysis is an effective approach to understanding the process of community formation. The phylogenetic tree of the species pool is reconstructed in the first step, and the phylogenetic tree obtained in the second step is used to analyze phylogenetic diversity. Sythetic trees have often been used in the construction of phylogenentic trees; however, in tropical rainforests with many closely related species, synthetic trees contain many unresolved nodes, which may affect the results of phylogenetic structure analysis. Here, we constructed a phylogenetic tree using DNA barcode sequences (*rbcL*, *matK*, *trnH‐psbA*) for 737 tree species from the rainforests of Borneo, which have a high‐species diversity and many closely related species. The phylogenetic tree had fewer polytomies and more branch length variations than the Phylocom synthetic trees. Comparison of community phylogenetic analyses indicated that values of the standardized effect size of mean pairwise distance (SES–MPD) were highly correlated between Phylocom and DNA barcode trees, but less so for the standardized effect size of mean nearest taxon distance (SES–MNTD), suggesting that caution is needed when using synthetic trees for communities containing many congeneric species, especially when using SES–MNTD. Simulation analysis suggested that spatial dependence on phylogenetic diversity is related to the phylogenetic signal of the species' habitat niche and the spatial structure of habitat, indicating the importance of detailed phylogeny in understanding community assembly processes.

## Graphical abstract

We determined DNA barcode sequences of 737 tree species in the Borneo rainforest, which has a high species diversity with many closely related species. Comparative analysis of community phylogeny suggested that caution should be exercised in the use of synthetic phylogenetic trees constructed with Phylocom and other tools for highly diverse forests. Simulation analysis was also conducted to demonstrate the significance of DNA barcode phylogeny in the study of community assembly processes.

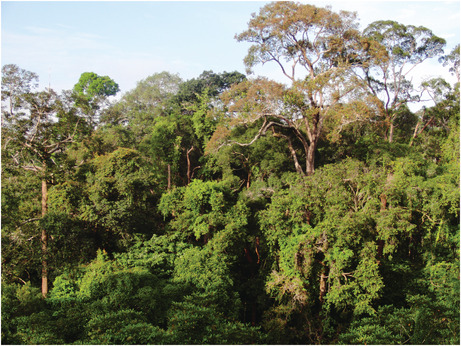

## 1 INTRODUCTION

Examining the phylogenetic structure of communities has been a widly used approach to understand the process of community assembly (McGill et al., 2006; Swenson, 2011, 2013, 2019; Webb et al., 2002). However, there are criticisms of inferring the ecological process of community assembly from patterns of phylogenetic diversity because different patterns of phylogenetic diversity can be caused by similar ecological processes (Gerhold et al., 2015; HilleRisLambers et al., 2012; Mayfield & Levine, 2010). For example, Mayfield and Levine (2010) argued that competitive exclusion of closely related species with similar niches can lead to both phylogenetic overdispersion and phylogenetic clustering and that large fitness differences and competitive exclusion can lead to phylogenetic clustering. Despite these critiques, exploring patterns of phylogenetic dispersion change in response to the environment is still valuable when combined with other information indicating that underlying environmental conditions likely influence community assembly (Cadotte & Tucker, 2017; Kraft et al., 2015).

There are two major steps in such analysis. The first step is to reconstruct a phylogenetic tree of the species pool, and the second step is to analyze the phylogenetic diversity of the target community using the resulting phylogenetic tree. In the second step, it is often useful to analyze how phylogenetic diversity varies with spatial scale because the relative importance of ecological processes can be inferred from the spatial scale dependence of phylogenetic diversity (Swenson, 2019).

For the first step, to infer the phylogenetic tree of the species pool, previous studies on plant communities (Hai et al., 2020; Li et al., 2019; Schweizer et al., 2017; Zappi et al., 2017; Zhao et al., 2020) have often used synthetic trees constructed by combining multiple existing phylogenetic data with software such as Phylomatic (Webb & Donoghue, 2005) and V. PhyloMaker (Jin & Qian, 2019). These trees usually contain many unresolved nodes and polytomies, and their branch length estimates are relatively coarse (Swenson, 2019). Recent studies in tropical rainforests have used DNA barcoding data to reconstruct phylogenies, which are more resolved than synthetic trees (Baraloto et al., 2012; Erickson et al., 2014; Kress et al., 2015). However, DNA barcoding trees may not resolve all polytomies, especially in the case of closely related species (Erickson et al., 2014; Swenson, 2019).

Unresolved nodes in the species pool phylogeny may affect the results of the second step, i.e., community phylogeny analysis. Therefore, several studies have examined how incomplete phylogenetic trees affect the results of community phylogenetic analysis (Kress et al., 2009; Li & Wiens, 2019; Patrick & Stevens, 2014; Pearse et al., 2013; Pei et al., 2011; Swenson, 2009). There is, however, a lack of comprehensive understanding about how incomplete phylogenetic trees affect the phylogenetic structure assessment, and how DNA barcode trees improve the analytical power in comparison to synthetic trees. In particular, the results from such assessments in species‐rich communities such as tropical rainforests are debatable. Although there are DNA barcode trees for tropical forests (Kress et al., 2009), they are still scarce in extremely species‐rich forests such as the lowland tropical rainforests of Borneo and the Amazon. To the best of our knowledge, Heckenhauer et al. (2017) were the only ones who examined the differences in the results of phylogenetic analysis using phylogenetic trees reconstructed with Phylomatic and DNA barcode data in a Bornean tropical forest. More empirical studies are needed to know the usefulness of DNA barcode trees in community phylogeny studies in the tropics.

It is often useful to compare results of community phylogenetic analyses at different spatial scales (Cavender‐Bares et al., 2006; Kembel & Hubbell, 2006; Swenson, 2019; Swenson et al., 2006, 2007). Previous studies have shown that the phylogenetic diversity of communities is often phylogenetically over dispersed at small spatial scales and clustered at larger spatial scales (Swenson, 2019). For example, Swenson et al. (2007) examined changes in phylogenetic diversity with spatial scale in five forests and found phylogenetic over dispersion at a small scale (25 m^2^) in three of the forests due to the exclusion of closely related species. At the larger scales >25 m^2^, phylogenetic clustering became stronger as the spatial scale increased in a Panamanian forest (Kembel & Hubbell, 2006; Pearse et al., 2013). However, further studies are necessary considering various forests, because spatial dependence may vary with the spatial structure of the environment and the presence or absence of phylogenetic signals in the species' habitat niche (Swenson, 2019).

In this study, we reconstructed phylogenetic trees using Phylomatic and DNA barcode data for a tropical rainforest in Borneo, one of the most species‐rich forests in the world (Davies et al., 2021). Subsequently, we (1) compared the resolution of the two phylogenetic trees and (2) compared the results of phylogenetic diversity analysis using Phylomatic and DNA barcode trees. We expect DNA barcode tree has higher resolution than Phylomatic tree in communities with many closely related species, leading to differences in phylogenetic diversity analysis. In addition, we (3) analyzed the relationship among plot size, habitat structure, and phylogenetic signal of species' habitat niche differences using simulations to examine the significance of multiple‐scale analysis in inferring community assemblage.

## 2 MATERIALS AND METHODS

### 2.1 Study site

**FIGURE 1:**
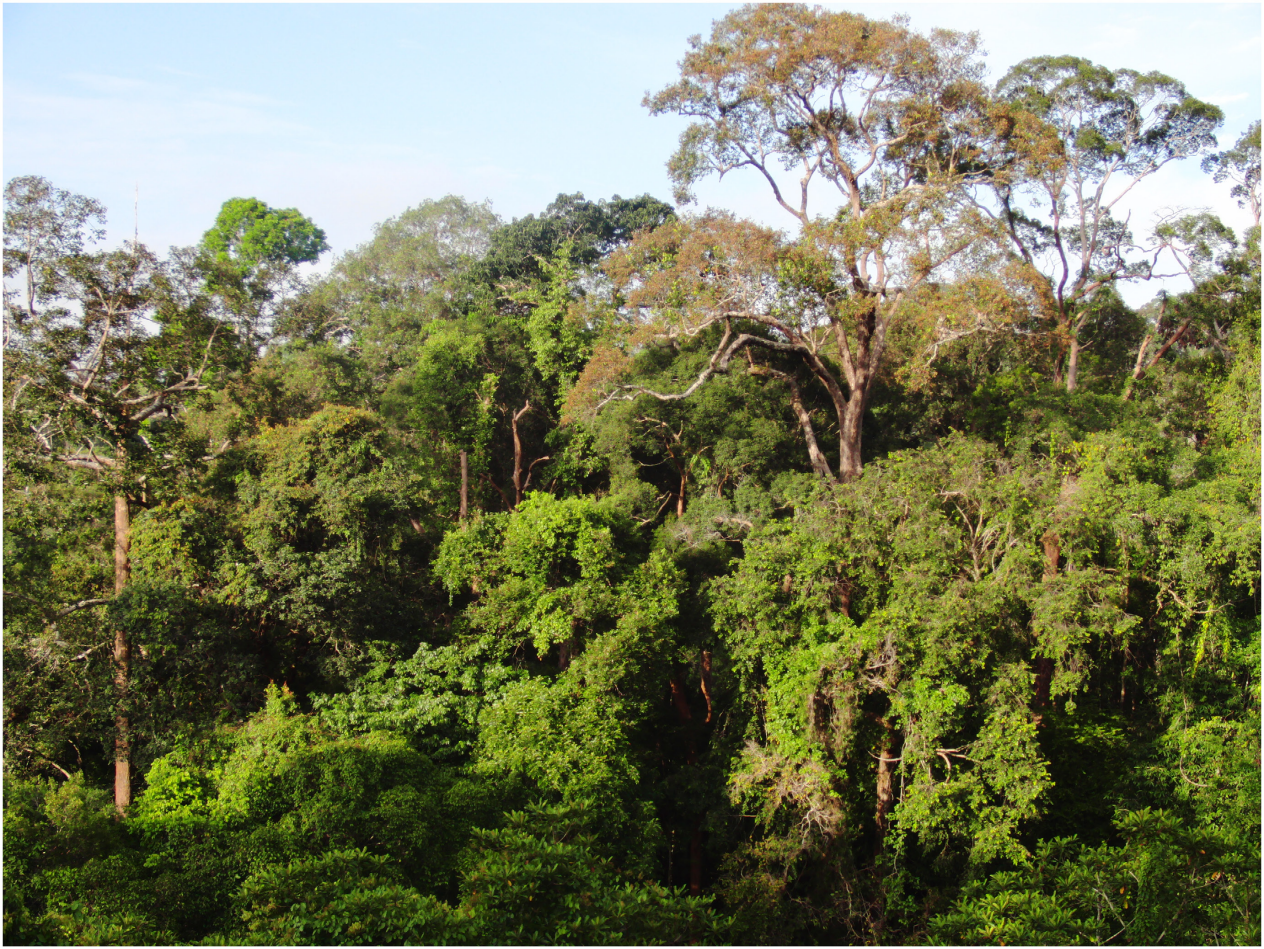
A view of the forest canopy in Lambir Hills national park. Photo taken from a canopy tower in the park.

**FIGURE 2:**
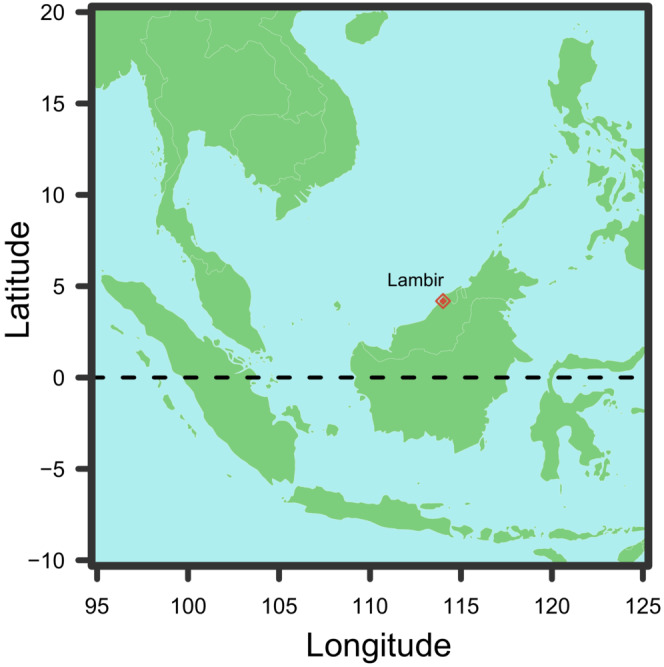
Location of Lambir Hills National Park. The map was created using R package “maps” (Becker & Wilks, 1993, 1995).

This study was conducted at the Lambir Hills Forest Dynamics plot (size: 1040 × 500 m, latitude: 4.1865, longitude: 114.0170, Figures 1 and 2) established in a tropical rainforest in Sarawak (Malaysian Borneo) in 1991 (Davies et al., 2021; Lee et al., 2004; Yamakura et al., 1995). Lambir has a humid tropical climate with no clear seasonality in temperature and precipitation. Average annual rainfall is 2725 mm (data from 1967 to 1998) at Miri Airport, approximately 20 km north of Lambir (Itoh et al., 2012; Lee et al., 2002). All the trees with a trunk diameter of 1 cm or more (approximately 400,000 individuals) were labeled, their locations were identified, the species are recorded, and the trunk diameters were measured (Lee et al., 2004; Yamakura et al., 1995). The survey of the trees has been conducted four times to date. The plot includes 1173 tree species (Lee et al., 2002). The higher tree diversity can be attributed to the heterogeneous topography and soil types in the plot (Davies et al., 2005).

### 2.2 Sampling and sequencing

From 2013 to 2017, we collected leaf samples from 1915 trees of 964 species in the study plot. Slingshots were used to collect leaves from tall trees. The leaf samples were dried at room temperature with silica gel. DNA was extracted from one sample of each species by a modified CTAB method. Three plastid regions (*rbcL*, *matK*, and *trnH*‐*psbA*) were amplified by PCR with several sets of primers (Table S2). The PCR products were sequenced using an ABI3730 Genetic Analyzer (Applied Biosystems) at Eurofins Genomics (Tokyo, Japan). The procedure was repeated for another sample of the same test species when valid sequences were not obtained. We used species for which at least the *rbcL* region was obtained for phylogenetic reconstruction.

### 2.3 Phylogenetic reconstruction

We constructed two phylogenies, hereafter referred to as “Phylomatic tree” and “DNA barcode tree”.

The Phylomatic tree was created using Phylomatic software (Webb & Donoghue, 2005) by pruning the angiosperm megatree R20160415.new (Gastauer et al., 2017) that is based on the APG IV (Angiosperm Phylogeny Group et al., 2016). The branch lengths were adjusted with “BLADJ” using a set of major node ages based on the node calibrations of Bell et al. (2010) (Table S3). All the procedures were performed using the R package “phylocomr” (Ooms et al., 2019).

The DNA barcode tree was reconstructed using a method that uses an existing megatree as the backbone and infers phylogenetic relationships within families by DNA barcoding sequences of *rbcL*, *matK*, and *psbA‐trnH*. Alignment was performed using ClustalW (Thompson et al., 1994) in MEGA7.0 (Kumar et al., 2016) for *rbcL* and MAFFT (Katoh, 2002) for *matK* and *psbA‐trnH*. Some sequences were manually corrected after alignment with the software. Phylogeny reconstruction was performed with RAxML Version 8.2.1 for Windows (Stamatakis, 2014). We used megatree R20160415.new (Gastauer et al., 2017) as the constraint tree to fix the relationships of families and deeper nodes because it is difficult to resolve deep nodes from DNA barcode sequences alone (Heckenhauer et al., 2017; Muscarella et al., 2014; Schreeg et al., 2010). While the best substitution model for *matK* and *psbA‐trnH* was GTR + Γ, GTR + I + Γ was best for *rbcL* based on AIC with ModelTest‐NG (Darriba et al., 2020). We used GTR + Γ for all loci because only one substitution model is allowed in RAxML Version 8.2.1. The evolutionary rates of *rbcL*, *matK*, and *psbA‐trnH* were independently estimated. We conducted 1000 rapid bootstrap replicates to evaluate clade support for RAxML phylogenetic reconstruction. We re‐analyzed the best model for each locus using RAxML‐NG (Kozlov et al., 2019), a later version of RAxML that supports multiple substitution models. With the exception of associations among very closely related species with low‐bootstrap confidence, the generated trees were largely comparable to one another (not shown). Therefore, we used the tree with GTR + Γ for all loci in subsequent analysis.

Divergence times were estimated with BEAST v2.6.6 (Bouckaert et al., 2019), in which the topology was fixed at the nodes with bootstrap values above 50 in the RAxML tree. Each partition was assigned an independent GTR + Γ substitution model with a Γ category count of 4. A log‐normal relaxed clock model (Drummond et al., 2006) was associated in common for all the partitions; and the Yule speciation model was selected. The prior ages of major orders and deeper nodes (*n* = 20) from Magallón et al. (2015) were used for calibration (Table S4). The 5 Markov chain Monte Carl (MCMC) chains were independently run for 75 million generations each and sampled every 10,000 generations. A consensus tree was obtained with TreeAnnotator v2.6.6, which is part of the BEAST 2 package, excluding the first 20% trees for each chain as burn‐in. Convergence of each independent runs was evaluated using tracer v1.7.2 (Rambaut et al., 2018), and the ESS of likelihood was confirmed to be 200 or more. Given the uncertainty of the DNAbarcode tree, we collapsed nodes with bootstrap values lower than 50% by RAxML (see File S1; Figures S1 and S2), and use the collapsed tree in all analyses of phylogenetic diversity below.

### 2.4 Phylogenetic diversity analysis

To evaluate the differences in phylogenetic diversity analysis, we calculated the correlation of phylogenetic diversity indices by using Phylomatic and DNAbarcode trees. There are various indices of phylogenetic diversity (Faith, 1992; Kembel, 2009; Webb et al., 2002). In this study, we used the standardized effect size of mean pairwise distance (SES–MPD) and the standardized effect size of mean nearest taxon distance (SES–MNTD) (Kembel, 2009). SES–MPD and SES–MNTD are essentially the same as the Net Relatedness Index (NRI) and Nearest Taxon Index (NTI), respectively, as described by Webb et al. (2002). We divided the 50 ha study plot into square plots of four different sizes: 10 × 10 m, 20 × 20 m, 50 × 50 m, and 100 × 100 m. We used “mpd.query” and “mntd.query” functions in the R package “phylomeasures” (Tsirogiannis & Sandel, 2016) to calculate SES–MPD and SES–MNTD, respectively.

To show that the results using Phylomatic and DNA barcode trees were different between similar communities, we analyzed how much difference in the phylogenetic diversity of two communities would make the results consistent regardless of the phylogenetic tree when comparing the phylogenetic diversity of the two communities. First, we compared the values of phylogenetic indices in all pairs of plots and examined whether the order of the values matched in Phylomatic and DNA barcode trees. Then, we estimated the probability of a match for plot pairs with different distances in phylogenetic diversity using logistic regression. The distance was defined as the mean of the two values calculated with Phylomatic and DNA barcode trees. We repeated the analysis for each plot size.

### 2.5 Relationship among plot size, habitat structure, and phylogenetic signal of species' habitat niche: Simulation analysis

We conducted simulations to reproduce the relationship between plot size and phylogenetic diversity indices obtained in this study and to demonstrate the effectiveness of multiscale analysis in estimating the process of community assembly. It examined the relationship between plot size and phylogenetic diversity indices for various habitat structures and phylogenetic signals of species' habitat.

**FIGURE 3:**
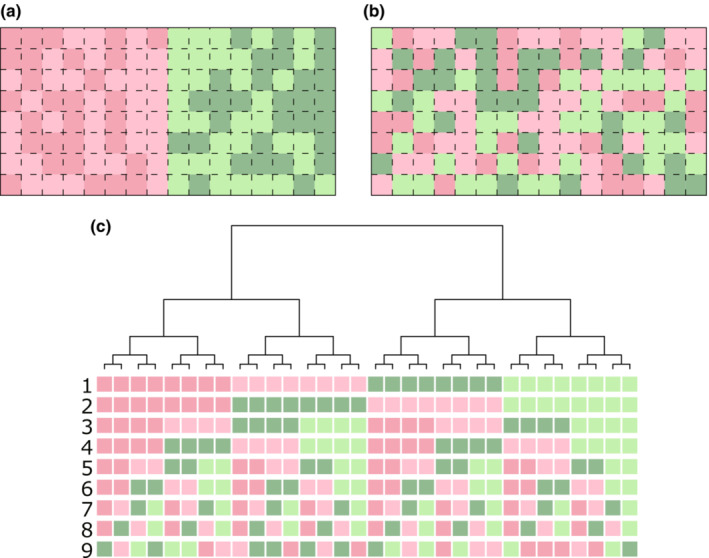
Setting in simulation analysis. (a and b) spatial structures of environmental types of two virtual plots. Color of each cell indicates environmental types. (a) the spatial structure is divided into two major areas with environmental types. (b) the spatial structure with disparate environmental types. (c) Phylogenetic structures of species' habitat niche. Color of each square indicates species' habitat niche corresponding to environmental type of the same color. There are 9 patterns of species' niche with various strength of phylogenetic signal.

Simulations were performed using a virtual plot of 8 × 16 cells (Figure 3a,b) and a fully resolved phylogenetic tree of 32 species (Figure 3c). Each cell in the plot was given one of the four environment types, and each species in the phylogenetic tree had one of the four species' habitat niches as well. We created two plots with different habitat structures: the first plot was divided into two areas with considerable different habitats and each area consisted of two similar habitat types distributed randomly (Figure 3a). The second plot consisted of four habitat types randomly distributed over the whole plot (Figure 3b). The habitat structure of the first plot was set to match the spatial distribution of the edaphic features of the study plot. The study plot is divided into two parts with distinct topography and soils: one with nutrient‐poor sandy soils on the upper slopes and ridges, and the other with nutrient‐rich clayey soils on the lower slopes and valleys (Davies et al., 2005; Russo et al., 2005; Tan et al., 2009). Within each part, the edaphic conditions vary marginally from place to place. Therefore, for simplicity, we assumed a plot with two areas with very different habitats, and randomly placed slightly different habitats in each area. The phylogenetic structure of species' habitat niche was designed with 9 patterns, which expected phylogenetic signals of different strengths: from phylogenetic clustering to phylogenetic overdispersion (Figure 3c). A phylogenetically random pattern was also added as a control.

**FIGURE 4:**
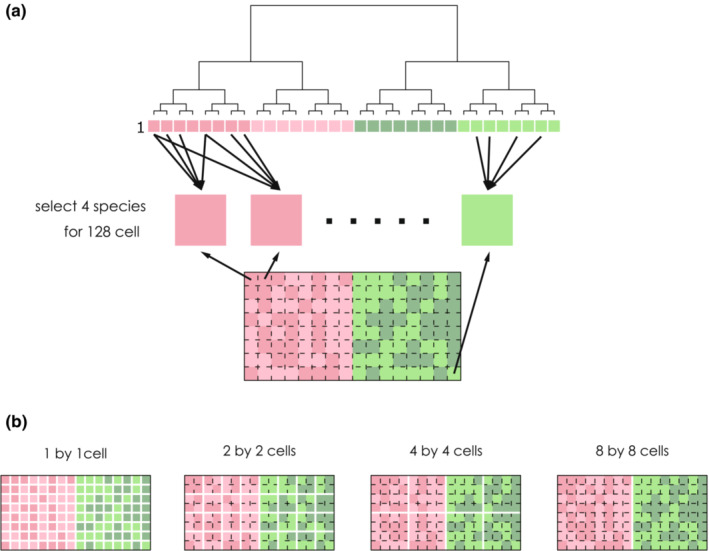
Images of simulations. (a) the process of creating a virtual community with the first row of the species’ habitat niche (number one in Figure 2c), and a structured habitat (Figure 2a). For each cell in the virtual plot, four species were randomly selected with a niche corresponding to the environment of the cell. (b) Separation images with different plot sizes in the virtual community. The plot was prepared in four different sizes: 1 × 1, 2 × 2, 4 × 4, and 8 × 8 cells.

We created a virtual community using a combination of two habitat structures and 9 niche patterns. For each cell in the virtual plot, we randomly selected four species with a niche corresponding to the environment of the cell (Figure 4a). We repeated this for all cells and obatined presence/absence data of species for 128 cells. Therefore, the same species could not be selected for each cell but could be selected for different cells that had the same environment. SES–MPD and SES–MNTD were then calculated using the virtual community for squares of four different sizes: 1 × 12 × 2, 4 × 4, and 8 × 8 cells (Figure 4b). The mean values of SES–MPD and SES–MNTD for each square size were then calculated. This procedure was repeated 100 times for each combination of the habitat structure and niche patterns.

## 3 RESULTS

### 3.1 Evaluation of DNA barcode tree

We obtained valid sequences of *rbcL*, *matK*, and *psbA*‐*trnH* regions for 737, 602, and 538 species, respectively (Table S1). We inferred the phylogenetic tree of 737 species, belonging to 224 genera, and 73 families (File S1; Figures S1 and S2). These values are 63%, 78%, and 93% of the species, genera, and families, respectively, recorded in the study plot (Lee et al., 2002). The 737 species represented 80% of the total number of ≥1 cm DBH (diameter at breast height) stems (*n* = 359,207) and 82% of the total basal area (2251 m^2^) of the 1997 census (Lee et al., 2002).

In the DNA barcode tree, 42%, 51%, and 65% of the nodes showed bootstrap supports (BS) ≥85%, ≥70%, and ≥50%, respectively. The DNA barcode tree determined some unresolved relationships between genera of families that were unresolved in the Phylomatic tree, e.g., Annonaceae, Dipterocarpaceae, Phyllantaceae, etc. (Table S1, Figures S1 and S2). The number of nodes with BS ≥50% was 476 in the DNA barcode tree. The variance in the log‐transformed branch length was greater for the DNA barcode tree (1.29) than for the Phylomatic tree (0.36), indicating that the DNA barcode tree contained more information on phylogenetic relationships.

### 3.2 Phylogenetic diversity analysis

**FIGURE 5:**
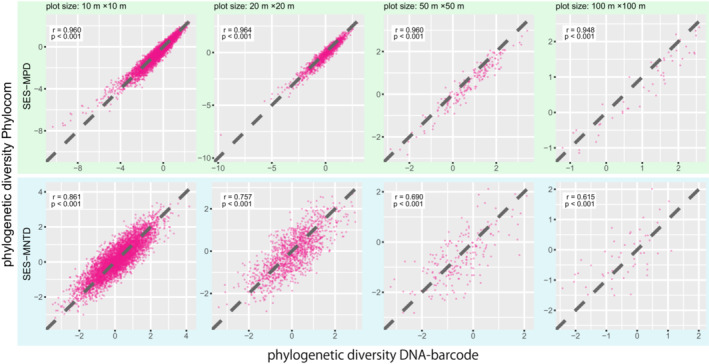
Correlation of phylogenetic diversity indices calculated with Phylomatic tree and DNA barcode tree for four plot sizes. The break line is a straight line with slope 1 passing through the origin.

The SES–MPD values calculated with Phylomatic and DNA barcode trees were highly correlated for all the plot sizes. However, the correlations of SES–MNTD values became weaker with the increase in plot size (Figure 5).

**FIGURE 6:**
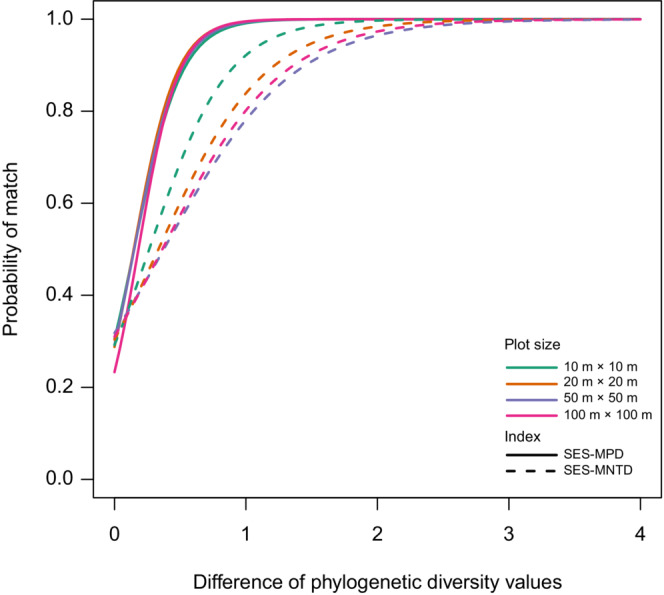
Relationships between the difference in phylogenetic diversity between plots and the probability of matching the rank of phylogenetic diversity values. Lines are logistic regression lines. The horizontal axis is the difference in phylogenetic diversity between the two plots. The vertical axis is the probability that plots with high‐phylogenetic diversity values match between the Phylomatic tree and the DNA barcode tree. Solid lines indicate SES–MPD, and dashed lines indicate SES–MNTD. Colors indicate plot size: Green 10 × 10 m^2^, orange 20 × 20 m^2^, purple 50 × 50 m^2^, and red 100 × 100 m^2^, respectively.

Logistic regression analysis revealed that the probability of match increased rapidly with increasing distance in phylogenetic diversity in SES–MPD and relatively slowly in SES–MNTD (Figure 6). No difference in the curve shape among different plot sizes in SES–MPD was noted, while the probability of a match in larger plots increased more slowly than that in smaller ones in SES–MNTD. When the difference in phylogenetic diversity was greater than 1, the probability of match was approximately 1 for SES–MPD. In contrast, for SES–MNTD, the difference of phylogenetic diversity was 1, the probability of match was 90% and 70% for 10 × 10 m and 100 × 100 m plots, respectively. This indicates that about 30% of 100 × 100 m plot pairs had different results between the Phylomatic and DNA‐barcode trees.

**FIGURE 7:**
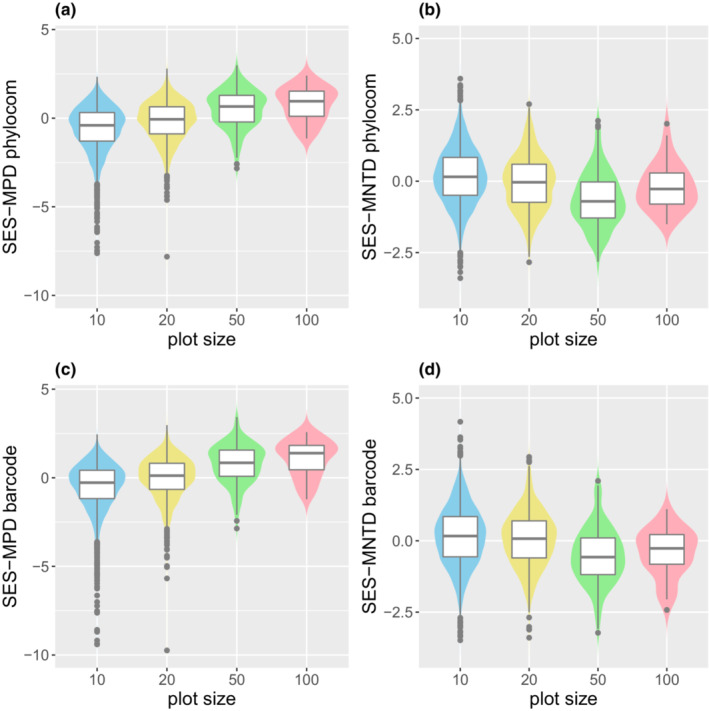
Phylogenetic diversity indices were calculated by Phylomatic (a and b) tree and DNA barcode (c and d) tree for four plot sizes.

The effects of plot size were similar in Phylomatic and DNA barcoding trees. The median of SES–MPD increased with increasing plot size from negative, indicating phylogenetic clustering, to positive, indicating phylogenetic overdispersion (Figure 7a,c; Table 1). In contrast, the median of SES–MNTD decreased with plot size from positive for 10 × 10 m plots to negative for bigger plots, although the median increased slightly from 50 × 50 m to 100 × 100 m plots (Figure 7b,d; Table 1).

**TABLE 1:** Community phylogenetic structure in quadrats at 4 spatial scales in the 52‐ha Forest dynamics plot on Lambir hills national park, Malaysia

Plot size	SES–MPD				SES–MNTD			
	Phylomatic		DNA barcode		Phylomatic		DNA barcode	
	median	*p*	median	*p*	median	*p*	median	*p*
10 × 10 m	−0.488	<.001	−0.374	<.001	0.163	<.001	0.154	<.001
20 × 20 m	−0.117	<.001	0.064	.057	−0.066	.020	0.058	.033
50 × 50 m	0.562	<.001	0.829	<.001	−0.668	<.001	−0.546	<.001
100 × 100 m	0.820	<.001	1.124	<.001	−0.263	.035	−0.371	.003

*Note*: SES–MPD and SES–MNTD are measures of phylogenetic structure using Phylomatic and DNA barcode trees. Positive SES–MPD and SES–MNTD values indicated phylogenetic overdispersion, whereas negative values indicate phylogenetic clustering. Significant *p*‐values indicate that the phylogenetic structure at a given spatial scale differed from zero according to the two‐tailed Wilcoxon test.

### 3.3 Relationship among plot size, habitat structure, and phylogenetic signal of species' habitat niche

**FIGURE 8:**
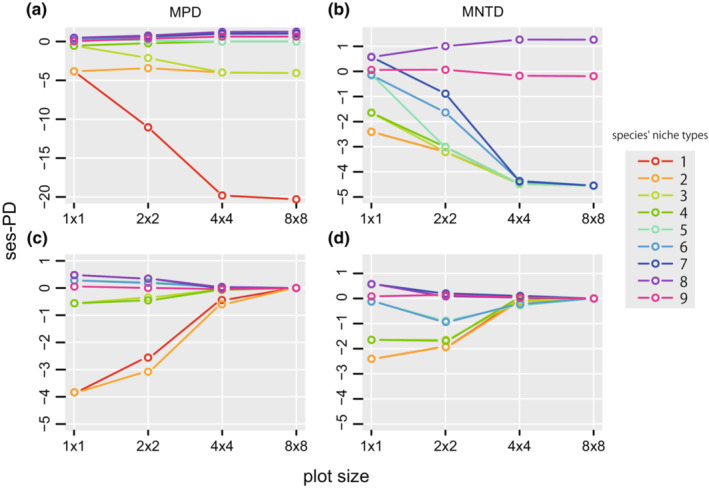
Changes in mean values of phylogenetic diversity indices with changes in plot size in simulations. The upper panel shows the results with the plot environment divided into two large plots as Figure 1a. The lower panel shows the results with the plot environment randomly arranged as Figure 1b. The colors of the lines indicate the respective sets of habitat niches in Figure 1c. In both MNTD plots, the line for set 1 overlap completely below the line for set 2.

The observed changes in the phylogenetic diversity indices with spatial scale (Figure 7) were reproduced in the simulations of the spatially structured habitats (Figure 8a,b) but not on the random habitat (Figure 8c,d). SES–MPD increased with increasing in plot size, whereas SES–MNTD decreased with increasing plot size both in the observed (Figure 7) and simulated results (Figure 8a,b). Moreover, the observed changes were reproduced only when the phylogenetic structure of the species' habitat niches was No. 6 or 7, the habitat niches of the most closely related species were the same or similar, and those of the second‐most related species were largely different, and these relationships were repeated in multiple clades (Figure 3c). Thus, phylogenetic signals are weak and habitat niches are phylogenetically over dispersed. These results suggest that spatially structured habitats and weak phylogenetic signal are required to produce observed changes.

## 4 DISCUSSION

### 4.1 Comparison of Phylomatic and DNA barcoding tree

This study demonstrates that the DNA barcoding tree, using existing megatrees as the backbone and inferring phylogenetic relationships within families by DNA barcoding data, reduced polytomy and increased variation in branch length compared to the Phylomatic tree. This indicates that DNA barcoding data provided more information on the differences in the phylogenetic distances between closely related species and genera. Although similar methods have been used in several previous studies (Erickson et al., 2014; Heckenhauer et al., 2017; Kress et al., 2010), this study shows its applicability to the Borneo rainforest, one of the most diverse forests in the world (Slik et al., 2015).

Nevertheless, our study also found that the phylogenetic relationship between closely related species could not be completely estimated by DNA barcoding data. Moreover, a similar observation has been reported in other studies (Heckenhauer et al., 2017). To estimate the phylogenetic relationships among closely related species, it is necessary to use more sequences. Fine phylogenetic relationships between closely related species were revealed by RAD‐seq in the Dipterocarpaceae of Southeast Asia (Heckenhauer et al., 2018) and Ebenaceae of New Caledonia (Paun et al., 2016); both families include many congeneric species. Combining the phylogenetic relationships of closely related species estimated by these methods using megatrees or DNA barcode trees would help reveal the detailed phylogenetic relationships of highly diverse tropical tree taxa. Other recent methods have been used for community phylogenetic analysis. For example, Jin et al. (2022) succeeded in improving the community phylogeny of subtropical forests by using widely sequenced plastid genomes. Targeted capture sequencing is also useful for analyzing relationships across wide phylogenetic scale (Brewer et al., 2019).

### 4.2 Comparison of phylogenetic diversity based on Phylomatic and DNA barcode trees

The results showed that the differences by phylogenetic trees were small for SES–MPD, regardless of the plot size. In contrast, the SES–MNTD differences were larger and increased with increasing plot size. These results are consistent with the previous studies that report SES–MPD is less sensitive to the resolution of the terminal node of the phylogenetic tree than SES–MNTD (Swenson, 2009). The higher differences in SES–MNTD in the larger plot size may relate to the number of species. In communities with a high number of species, SES–MNTD differs between resolved and unresolved trees (Swenson, 2009). This is because, differences in the number of polytomies and the number of species included in each polytomy between Phylomtic and DNA‐barcode trees increased with the number of species in a community. The number of polytomies is large and many species are included in the polytomies in the Phylomatic tree, especially in species‐rich communities. As SES–MNTD refers to the distances between the most closely related species, it is largely affected by the degree of resolution of such species pairs. At the study site, the average numbers of species in each plot were 36.9 and 450.1 for 10 × 10 m and 100 × 100 m plots, respectively. Moreover, the average numbers of species in the most species‐rich genera, which are likely to be polytomy in the Phylomatic tree were 2.9 and 23.6 for 10 × 10 m and 100 × 100 m plots, respectively.

The SES–MPD was less affected by the difference in phylogenies than SES–MNTD when comparing phylogenetic diversity of two communities (Figure 6). This indicates that we can use SES–MPD except when discussing small differences in phylogenetic diversity. However, when using SES–MNTD, care should be taken while interpreting the results using the Phylomatic tree, especially for large communities with many species. In this study, the probability of mismatch in results between the Phylomatic and DNA‐barcode trees was ca. 30% between communities with a difference in phylogenetic diversity of ca.1 for 100 × 100 m plots (Figure 6). The difference in phylogenetic diversity of less than one has often been observed at a local scale (< 1 km) in tropical forests. In this study, the mean difference in SES–MNTD for 100 × 100 m (1 ha) plots was 0.94. In a 50‐ha tropical forest plot on Barro Colorado Island (BCI) in Panama, the differences in SES–MNTD between seven communities divided based on soils (1.2–25 ha) were 0.01 to 0.76 (Kembel & Hubbell, 2006). In a 20‐ha plot in a subtropical forest in China, the differences in SES–MNTD between the five communities with varying habitat types (2.5–6.9 ha) ranged from 0 to 0.87 (Pei et al., 2011). Therefore, in the case of many closely related species, such as tropical rainforests, it would be better to use a higher resolution phylogenetic tree rather than a synthetic tree such as Phylomatic when examining small differences in phylogenetic diversity among similar communities.

### 4.3 Effects of plot size on phylogenetic diversity

The scale dependence of phylogenetic diversity in Borneo obtained in this study differed from previous studies in Panama (Kembel & Hubbell, 2006; Pearse et al., 2013). In Borneo, the phylogenetic structure changed from clustering to overdispersion with an increase in plot size in SES–MPD (Figure 7a,c). In contrast, in SES–MNTD, the phylogenetic structure changed from overdispersion to clustering (Figure 7b,d). However, in a 50‐ha plot on BCI, the phylogenetic structure became more clustered with increasing plot size in both SES–MPD and SES–MNTD (Kembel & Hubbell, 2006). The differences in scale dependence between Borneo and Panama can be explained by differences in the phylogenetic relationships of species' habitat niches and the spatial structure of habitats within the plot, as explained later.

The observed changes in the phylogenetic diversity indices with spatial scale in the Lambir community (Figure 7) were reproduced in the simulations only when the phylogenetic signal of habitat niches was weak and habitats were spatially structured (Figure 8a,b). When species in the phylogeny with weak habitat signals (pattern 6 or 7 in Figure 3c) are distributed in structured habitats (Figure 3a), in which the area of the same or similar habitat increases as the plot size increases, the probability of both the most closely and most distantly related species pairs in the same plot increases more than expected from randomly selected species pairs. As a result, changes in SES–MNTD and SES–MPD differed depending on the degree of influence of the probabilities of closely and distantly related species. SES–MNTD decreases with plot size because it is influenced more by the presence of closely related species than that by the distantly related ones (Swenson, 2019). In contrast, as SES–MPD is influenced more by phylogenetically distant species (Swenson, 2019), SES–MPD increases with the plot size.

The changes in phylogenetic diversity indices observed in BCI, that both SES–MPD and SES–MNTD decreased with plot size were similar to the simulation results with the phylogenetic structure of the species' habitat niches No. 1 or 3 (Figure 3c) and the habitat structure (Figure 3a). In the phylogenetic structure of habitat niches No. 1 and 3, species with the same or similar habitats are phylogenetically clustered, i.e., having a phylogenetic signal. When the phylogenetic signal is present, the probability of the presence of phylogenetically close species pairs increases as the plot size increases, but the probability of the presence of phylogenetically distant species pairs is not higher than expected from the random selection of species pairs independent to plot size. Therefore, both SES–MNTD and SES–MPD decrease with plot size because values are determined only by the probability of closely related species pairs if the probabilities of phylogenetically distant species pairs are the same.

Changes in phylogenetic diversity indices with plot size were also affected by the habitat structure. When the spatial structure of the habitats was random (Figure 3b), the dependence on plot size varied with the strength of the phylogenetic signal in the habitat niche. In a random habitat structure, the probability that a plot includes all kinds of habitats increases with plot size. As there are limited kinds of habitats in a small plot, the number of phylogenetically close species increases if there is a strong phylogenetic signal in the habitat niche, and the number of phylogenetically distant species increases if there is phylogenetic over dispersion in the habitat niche. Therefore, SES–MPD and SES–MNTD have negative values at smaller plots when the phylogenetic signal is strong and have positive values when the habitat niche is phylogenetically over dispersed (Figure 7c,d). SES–MPD and SES–MNTD moved toward zero at larger plots independent of the strength of the phylogenetic signal as the larger plots contain all kinds of habitats.

The habitat and niche structures used in the simulations were roughly consistent with the observed ones in Lambir and BCI. Both 50‐ha plots are spatially structured and divided into several major environments (Davies et al., 2005; Harms et al., 2001; Russo et al., 2005). Phylogenetic signal in habitat niche is stronger in BCI than in Lambir. In BCI, most species were distributed non‐randomly concerning soil (Harms et al., 2001), and congeneric species pairs had similar habitat niches than non‐congeneric pairs (Baldeck et al., 2013), suggesting a strong phylogenetic signal. Contrastingly, congeneric species in Lambir had different habitat niches in many genera, e.g., *Aporosa* in family Phyllantaceae (Debski et al., 2002), *Dryobalanops* in Dipterocarpaceae (Itoh et al., 2003), *Scaphium* in Malvaceae (Yamada et al., 1997), *Macaranga* in Euphorbiaceae (Davies et al., 1998), and *Ficus* in Moraceae (Harrison et al., 2003). Some species of the different genera coexisted in the same habitat, suggesting that there is overdispersion in habitat niches in Lambir trees. It is not clear yet whether the habitat niches of the most closely related species are similar within a species rich genus in Lambir, but the current study suggests this may be the case. Nevertheless, more detailed phylogeny is needed to confirm this.

One limitation of our simulation is that we assumed that the species distributions were determined only by habitat. Rather, species distributions are determined by a variety of factors, including interspecific competition, disease, and predation as well as habitat (Connell, 1971; Gause, 1934; Holt et al., 1994; Janzen, 1970). Further research is needed on the factors that determine species distribution to evaluate the generality of the importance of habitat niche in community assemble.

## 5 CONCLUSIONS

We reconstructed a phylogenetic tree with a higher resolution than the synthetic tree for the extremely species‐rich tropical rainforests of Borneo using DNA barcode sequences. A comparison of community phylogenetic analyses suggested that caution should be exercised when using synthetic trees for communities containing many congeneric species, especially when using the SES–MNTD. The simulations suggest that examining the phylogenetic diversity of communities at different spatial scales provides useful information about the phylogenetic structure of habitat niches and the spatial structure of habitats, which is useful for understanding community assembly processes. However, DNA barcode sequences cannot completely resolve the phylogeny of genera containing extremely large numbers of species. More informative methods, such as RADseq, plastid genome sequencing, and target capture sequencing, should be used to infer the phylogenetic relationships for such genera.

## AUTHOR CONTRIBUTIONS

**Seiya Okuno:** Conceptualization (equal); data curation (equal); formal analysis (equal); investigation (equal); methodology (lead); resources (equal); visualization (lead); writing – original draft (equal); writing – review and editing (equal). **Tingting Yin:** Data curation (equal); formal analysis (equal); investigation (equal); resources (equal). **Satoshi Nanami:** Conceptualization (equal); formal analysis (equal); funding acquisition (supporting); investigation (equal); writing – review and editing (equal). **Shuhei Matsuyama:** Data curation (equal); investigation (equal); resources (equal); writing – review and editing (equal). **Koichi Kamiya:** Data curation (equal); investigation (equal); writing – review and editing (equal). **Sylvester Tan:** Investigation (equal); resources (equal); writing – review and editing (equal). **Stuart Davies:** Funding acquisition (lead); project administration (equal); supervision (equal); writing – review and editing (equal). **Mohizah Mohamad:** Data curation (equal); project administration (equal); supervision (equal); writing – review and editing (equal). **Takuo Yamakura:** Funding acquisition (equal); project administration (equal); supervision (equal); writing – review and editing (equal). **Akira Itoh:** Conceptualization (equal); formal analysis (equal); funding acquisition (lead); methodology (supporting); project administration (equal); supervision (equal); visualization (supporting); writing – original draft (equal); writing – review and editing (equal).

## CONFLICT OF INTEREST

The authors declare no conflicts of interest.

## Supporting information


Table S1.

Table S2.

Table S3.

Table S4.

Figure S1.

File S1.

## Data Availability

Sequences are available in GenBank (see Supporting Information Table S1). Plot tree data are available upon request at the ForestGEO online portal: http://www.forestgeo.si.edu.
