# Supplementary material for: Community phylogeny and spatial scale affect phylogenetic diversity metrics in a species‐rich rainforest in Borneo
Source: Ecol Evol. 2022 Nov 22;12(11):e9536. doi: 10.1002/ece3.9536 (PMC9682210; doi:10.1002/ece3.9536)
Supplement: Supplementary file 5 — Figure S1. [file ECE3-12-e9536-s006.pdf]

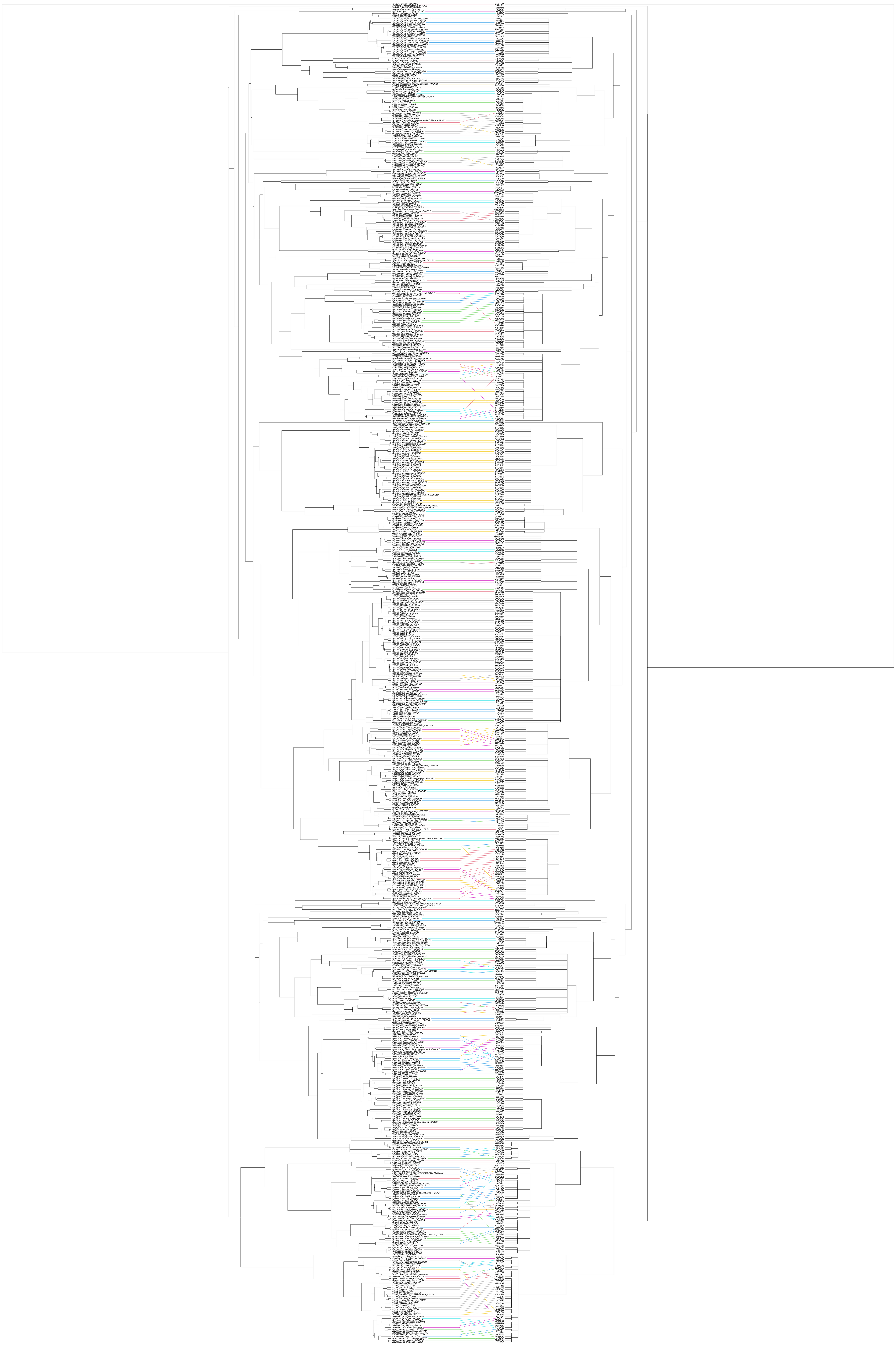

Figure S1. DNA barcode tree (left) and Phylomatic tree (right). The colors of the lines indicate genera.

DNA barcode (collapced) | Phylomatic

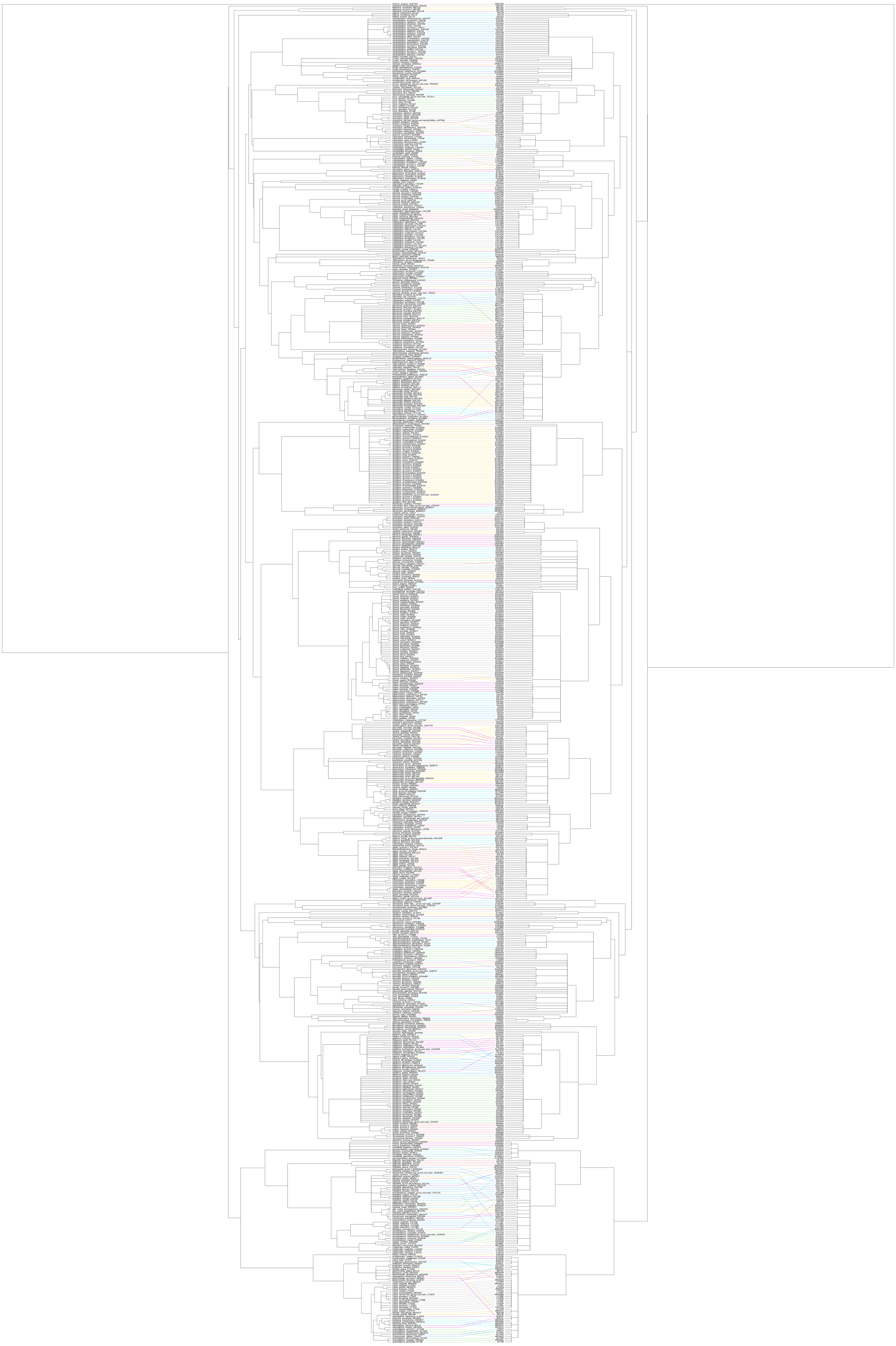

Figure S2. DNA barcode tree (left) and Phylomatic tree (right). Bootstrap values < 50 % were collapsed to polytomies. The colors of the lines indicate genera.
